# Retraction: Comprehensive N-glycan profiles of hepatocellular carcinoma reveal association of fucosylation with tumor progression and regulation of FUT8 by microRNAs

**DOI:** 10.18632/oncotarget.28788

**Published:** 2025-12-31

**Authors:** Lei Cheng, Shuhang Gao, Xiaobo Song, Weijie Dong, Huimin Zhou, Lifen Zhao, Li Jia

**Affiliations:** ^1^College of Laboratory Medicine, Dalian Medical University, Dalian 116044, Liaoning Province, China; ^2^Department of Laparoscopic Surgery, The First Affiliated Hospital of Dalian Medical University, Dalian 116011, Liaoning Province, China; ^3^Department of Oncology, The First Affiliated Hospital of Dalian Medical University, Dalian 116011, Liaoning Province, China; ^4^Department of Medical Biology, Faculty of Health Sciences, University of Tromsø, Tromsø, Norway; ^5^Department of Biochemistry, Dalian Medical University, Dalian 116044, Liaoning Province, China; ^6^Department of Microbiology, Dalian Medical University, Dalian 116044, Liaoning Province, China; ^*^These authors contributed equally to this work

**This article has been retracted:** Oncotarget has completed its investigation of this paper following feedback from our readers. We subsequently discovered that the corresponding author, Jia Li was investigated by the National Natural Science Foundation of China (NSFC) for image duplication and rotation across 15 papers, including this submission to Oncotarget. As a result of their investigation, Jia Li’s qualification to apply for NSFC projects was cancelled for 5 years (July 20, 2021, to July 19, 2026), she was criticized in a circular, and four of her NSFC projects had their funds withdrawn and project approval numbers revoked. Based on the findings of these investigations, the Oncotarget Editors have decided to retract the article.

Original article: Oncotarget. 2016; 7:61199–61214. 61199-61214. https://doi.org/10.18632/oncotarget.11284

